# Divergence between facial expressions and self-reported emotions: Sex differences in responses to video-based emotional stimuli

**DOI:** 10.1371/journal.pone.0352759

**Published:** 2026-06-29

**Authors:** Sunyoung Choi, Changjin Jung, Jieun Kim, Sungsil Ko, Jiyoun Choi, Hyungjun Kim

**Affiliations:** 1 NeuroGlymph Imaging and Modulation Center, Korea Institute of Oriental Medicine, Daejeon, South Korea; 2 Department of Social Psychology, Sookmyung Women’s University, Seoul, South Korea; Universita degli Studi di Pisa, ITALY

## Abstract

Emotional responses involve multiple components, including subjective experience and behavioral expression, which do not always align. Moreover, sex differences in emotional processing appear to vary across emotion types and response modalities. This study investigated sex differences and concordance between self-reported emotional experience and facial expressions elicited by naturalistic emotional video stimuli in a Korean adult sample. One hundred forty-eight healthy adults viewed video clips inducing joy, anxiety, sadness, and neutral states; spontaneous facial expressions were recorded and analyzed using the iMotions automated facial expression analysis software. After watching the stimuli, participants rated their subjective emotional experiences on discrete emotion scales (joy, sadness, disgust, fear/anxiety) and affective dimension scales (valence and arousal). Facial expression measures were summarized as positive and negative expressions. Age effects were statistically controlled for both facial expression and self-rating measures, and non-parametric analyses were conducted on the age-adjusted values because most variables deviated from normality. The video stimuli reliably elicited their intended target emotions in both self-reported ratings and facial expression patterns. Females exhibited stronger positive facial expressions than males during joyful stimuli despite comparable self-reported joy ratings. For anxiety stimuli, females showed higher fear/anxiety ratings and stronger negative facial expressions, whereas for sadness stimuli, females reported higher sadness ratings without corresponding differences in facial expressions. Females also reported higher arousal than males for negative stimuli. Concordance between facial expressions and self-reported ratings varied by emotion and sex, with robust associations for joy across both sexes, weaker or absent associations for sadness, and sex-specific associations for anxiety. These findings demonstrate that sex differences in emotional responding are emotion-specific and component-specific, highlighting partial dissociations between subjective experience and expressive behavior. This study provides culturally specific validation of dynamic video-based emotion paradigms and supports continuous facial expression analysis as an objective complement to self-report measures in Korean populations.

## Introduction

Emotion is not a simple phenomenon but a complex and dynamic process, characterized by the interaction of multiple components, including subjective experience and behavioral expression [1–5]. Subjective experience refers to the internal emotional state that an individual can consciously experience and describe (e.g., feeling happy), whereas behavioral emotional expression involves external motor reactions that manifest emotion, such as facial movements.

Facial emotional expressions have long been recognized as a critical channel for conveying internal states, beginning with Darwin’s seminal work on emotional expression [6], but facial expressions do not always covary with what is internally felt [7–11]. For instance, Franěk et al. (2022) [8] found that self-reported positive emotions varied as a function of stimulus type, whereas facial expression magnitudes remained relatively stable, demonstrating a decoupling between subjective and behavioral components. Similarly, Zempelin et al. (2021) [11] reported that subjective sadness ratings increased after viewing sadness-inducing film clips, without a corresponding increase in facial expression intensity for sadness. Thus, a comprehensive understanding of emotion requires an integrative approach that considers multiple components of emotion processing.

In addition to variability across emotional components, another source of complexity in emotional processing lies in sex differences. Although it is commonly assumed that women are more emotional than men, empirical findings on sex differences in emotional processes have been mixed [12–14]. For instance, Saylik et al. (2018) [14] explored sex differences in emotional experience by asking participants to identify emotional faces as one of six emotions. Their results showed that females responded faster than males, whereas no sex difference was observed in the identification accuracy. Extending the investigation to expressive and experiential components of emotion, Kring and Gordon (1998) [12] examined facial expressions and self-reported emotional experiences elicited by emotion-inducing film clips. They found that women exhibited facial expressions more frequently than men, but self-reported ratings did not differ between women and men, suggesting that sex differences in emotion are not uniform but may depend on the specific emotional component under investigation. Moreover, the type of emotion appears to moderate sex-related differences, with some emotions showing larger variation between sexes than other emotions [15–19], indicating that emotion type should be explicitly considered when examining sex differences in emotional functioning.

In the present study, we examined sex differences in both subjective experiences and external expressions by assessing self-reported emotional states and facial behaviors. Prior studies have often examined these components in isolation in the context of sex differences [18,20], or have been limited by small sample sizes [12]. To address this gap, the current study investigated the integration of subjective experience and facial expression using a relatively large sample (N = 158; 148 after preprocessing). By assessing both components simultaneously, our study provides a more comprehensive evaluation of whether sex-related differences are consistent across both domains or specific to one component.

We further systematically examined whether and how the sex differences and concordances among subjective and behavioral components were modulated by different emotion types. To this end, we employed video clips designed to elicit a range of emotional states—specifically, joy, anxiety, sadness, and neutrality—and analyzed the results separately for each emotion. This study contributes to existing knowledge on emotion processing by addressing whether sex-related patterns are emotion-specific or generalizable across emotion categories.

This study focused specifically on facial emotional expressions in a Korean sample. Numerous empirical studies, particularly by Ekman and colleagues, established the universality of facial expressions in conveying and recognizing certain emotions [21,22]. However, a substantial body of cross-cultural studies has demonstrated that cultural and regional background influences the expression and perception of facial emotions (for a meta-analysis, see [23,24]). Given such cultural variability, findings based on a Korean sample contribute to a more comprehensive understanding of cultural variation in facial emotional expressions. Moreover, by examining sex differences in this cultural context, our study extends existing knowledge on how cultural factors may interact with sex-related emotional patterns.

In sum, the present study aims to examine sex differences in subjective emotional experience and facial expression in response to emotionally evocative video stimuli in a Korean population. Using self-report ratings alongside facial expression data analyzed via an analysis tool (iMotions), we investigated differences between males and females in both self-report ratings and facial expressions across emotion types, as well as within-group concordance between the self-report ratings and facial expressions. This approach aims to provide a more refined understanding of emotional processing across sexes in a relatively understudied population.

## Methods

### Participants

This study recruited 158 healthy adult participants (mean age = 35.7[age range: 19−59], SD = 10.68, Male[N]=79) with no history of psychiatric or neurological disorders. All participants underwent a screening process prior to enrollment; inclusion required a Beck Depression Inventory (BDI) score below 14 and a Beck Anxiety Inventory (BAI) score below 9. As shown in Table 1, there were no significant sex differences in age (t[146] = −0.35, p = 0.727), years of education (t[146] = −0.188, p = 0.851), BAI (t[145] = −1.321, p = 0.189) and BDI (t[137.66] = −0.951, p = 0.343) scores. The study protocol was approved by the Institutional Review Board (IRB) of Sookmyung Women’s University (SMWU-2203-HR-015), and registered in the Clinical Research Information Service, established by the Korea Centers for Disease Control and Prevention and embodied as a part of the Primary Registries in the World Health Organization International Clinical Trials Registry Platform (KCT0008724, https://cris.nih.go.kr). Participants were recruited from 18 April 2022–17 April 2023. Written informed consent was obtained from all participants prior to participation.

**Table 1:** Statistics for comparison of male and female participants on demographic and clinical characteristics.

	Males (N = 75)		Females (N = 73)		Statistics	
	Mean	SD	Mean	SD	t value	p value
Age	35.64	10.51	36.26	11.02	−0.350	0.727
Education(year)	17.08	2.05	17.14	1.61	−0.188	0.851
BAI	2.22	2.96	2.96	3.81	−1.321	0.189
BDI	3.17	2.89	3.68	3.61	−0.951	0.343

### Procedure

Facial expression movements were captured at a rate of 30 Hz using a webcam while participants viewed emotional video stimuli presented on the laptop display. Participants were seated approximately 50 cm away from a laptop screen measuring 34.0 × 20.3 cm², corresponding to visual angles of 37.6° horizontally and 22.6° vertically, and wore headphones. During the facial expression recording, participants were left alone and were instructed to experience the emotions naturally while viewing the stimuli.

The experiment employed a block design format, comprising 30-second emotional video segments followed by a 10-second fixation cross. Each block contained two video clips for each of joy, sadness, and neutral categories, and three clips for the anxiety category. The stimuli were naturalistic audio-visual clips obtained from comedy shows, news broadcasts, and publicly available YouTube contents. Four blocks were shown for each stimulus emotion category (joy, sadness, anxiety, and neutral), yielding 16 blocks in total (for the details, see S1 Fig in S1 File). The 16 blocks were divided into two sections of eight blocks each. The block order within each section was fixed across participants, whereas the order of the two sections was counterbalanced. Each section began with a 30-s fixation cross, and participants were allowed to take sufficient breaks between sections. The block sequences for section 1 and 2 are shown in S1 Fig in S1 File.

At the end of each section, self-reported emotional ratings for the previously viewed videos were collected using E-Prime 3.0 software. Participants viewed screenshots representing each video clip and provided two types of self-ratings: category-specific discrete emotion self-ratings and affective dimension ratings. For discrete emotion ratings, participants rated the extent to which they experienced each emotion (joy, sadness, disgust, and fear/anxiety), selected to correspond to the stimulus categories and the facial expression measures, using a 10-point scale (0 = not at all, 9 = extremely). Affective dimension ratings were obtained using two 9-point Likert scales assessing arousal (perceived intensity/activation; 1 = calm, 9 = excited) and valence (pleasant–unpleasant continuum; 1 = negative, 9 = positive).

### Data preprocessing

#### Facial expression data.

The iMotions 7.0 software (iMotions Inc., MA, USA) was used to automatically extract facial features from the recorded facial videos. This software estimated probabilistic scores of 17 distinct facial Action Units (AUs; e.g., brow raise, brow furrow, eye widen, cheek raise, etc.; see S1 Table in S1 File) based on the Facial Action Coding System (FACS) [25]. Facial video recordings of all 158 participants were reviewed to ensure data quality and reliability. Three participants who showed signs of drowsiness or frequently obscured face with their hands—making accurate facial expression analysis unreliable—were excluded from the analysis. Seven additional participants were excluded due to abnormally high average AU values (>30; AU range: 0–100) across the recoding, likely reflecting failures in facial feature tracking or atypical facial postures that compromised data validity.

To address these high values, manual verification was performed for all cases exceeding the threshold of 30. This process identified instances where facial morphology or cosmetic factors—such as eyebrow shape or makeup—artificially inflated inner brow raises, leading to misclassification as sadness. Similarly, naturally large eyes resulting in apparent eye widening (fear classification) and baseline lip morphology interpreted as upper lip raises (disgust classification) were detected. Following the exclusion of these misclassified cases, data from 148 participants were included in the final analysis.

The probabilistic scores for seven basic emotional expressions—joy, sadness, disgust, fear, anger, surprise, and contempt were obtained from the iMotions output. In our analysis, joy, sadness, fear and disgust expression scores were selected: Joy and sadness scores directly corresponded to the joy and sadness stimulus conditions; fear scores were used as an index of anxiety-related facial responses, given the conceptual overlap between anxiety and fear; and disgust expression scores were additionally included to capture potential facial responses elicited by the stimuli. These expression values were extracted at a sampling rate of 30 frames per second and were averaged over the entire duration of each stimulus presentation.

To evaluate the reliability of facial expression measurements obtained via iMotions software, an intraclass correlation coefficient (ICC) analysis was conducted. The software quantified facial expression scores for each emotion on a continuous scale ranging from 0 to 100, based on predefined combinations of AUs required to express each emotion. For human ratings, an experienced rater (J.C.) independently evaluated 94 captured still frames representing five emotional expressions (joy, sadness, fear, disgust, and surprise) from 148 participants using a 5-point scale. Surprise expressions were included in addition to the four target emotions to assess the general reliability of facial expression measurement beyond the stimulus-specific categories.

Because the scoring scales of the human ratings and the iMotions outputs differed, both sets of values were z-standardized prior to ICC computation. ICCs (model 1,2) and their 95% confidence intervals were calculated for each expression type, with the results summarized in S2 Table in S1 File. The analysis revealed that joy expressions showed the highest agreement between iMotions and the rater J.C. (ICC = 0.94), whereas the remaining expressions exhibited moderate agreement, with ICC values in the 0.70 range.

Previous research (see [26] for an extensive review) has shown that facial movements alone do not reliably map onto discrete emotion categories and are often better characterized at a broader affective level. In line with this perspective, the present study focused on emotional valence (positive and negative) rather than discrete facial emotion categories. Accordingly, positive facial expression was defined as the iMotions-derived joy score, whereas negative facial expression was defined as the maximum value among the iMotions-derived sadness, fear, and disgust scores at each frame, reflecting the presence of any negative facial signal.

This valence-based approach was further supported by our reliability analysis. Specifically, agreement between the iMotions outputs and the human rater was higher when facial expressions were analyzed at the valence-level (ICC = 0.85) than when individual negative facial expressions were considered separately (ICC ≤ 0.74). Taken together, both the theoretical considerations outlined by [26] and the improved reliability of the valence-based measure motivated our focus on facial expressions primarily at the level of emotional valence.

#### Age adjustment and residualization of emotional variables.

Previous studies have shown that age influences both emotional expression and emotion recognition [27–31]. For example, compared to younger adults, older adults exhibit different expressive patterns and use more facial muscles overall, and they differ in emotion recognition accuracy for certain stimulus types and emotion categories. Accordingly, age was statistically controlled in the analyses of facial expression and self-reported ratings.

For facial expression data from iMotions, the log-transformed values (log10(1 + x)) were used to correct for skewness in the original 0–100 scale; other variables underwent age correction without prior transformation. Age effects were controlled by regressing each variable of interest on age and using the standardized residuals (mean = 0, SD = 1). The standardized residuals were transformed back to the original scale by multiplying by the variable’s standard deviation and adding the original mean:


$$\begin{array}{c} A=\overset{―}{Y}+\left(Z_{RE\text{1}}\times s_{Y}\right) \end{array}$$

where $\overset{―}{Y}$ is the original mean of 𝑌, $s_{Y}$ is the standard deviation of 𝑌, and $Z_{RE\text{1}}$ is the standardized residual. These age-controlled variables were used in all subsequent analyses (see the S3 Table in S1 File).

## Results

### Normality assessment

Prior to the main analyses, the normality of all age-controlled variables—including self-reported ratings (joy, sadness, disgust, and fear/anxiety, arousal, and valence ratings), and iMotions-derived facial expression data (positive and negative facial expressions)—was examined using the one-sample Kolmogorov–Smirnov (K–S) test with a significance level of α = 0.05. Of the 32 variables examined, only the valence ratings for the joy stimulus met the normality assumption; the remaining 31 variables significantly deviated from a normal distribution (see S4 Table in S1 File). Given these results, all subsequent analyses were performed using non-parametric statistical methods for methodological consistency. Accordingly, descriptive statistics are reported as medians and interquartile ranges (IQRs), rather than means and standard deviations, to appropriately reflect the non-normal distributions of the data.

### Predominant emotional responses by stimulus condition

For each stimulus emotion category, predominant emotional responses were identified separately for each response type (facial expression, and discrete emotion ratings) based on the highest median value and compared using the Wilcoxon signed-rank test. Effect sizes r was calculated as $r=\frac{Z}{\sqrt{N}}$.

**Fig 1:**
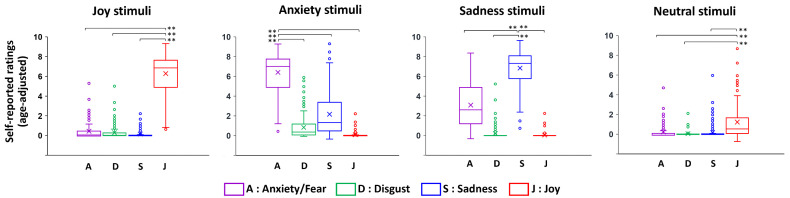
Boxplots showing medians, interquartile ranges, and individual data points for self-reported ratings of discrete emotions (fear/anxiety, disgust, sadness and joy) across stimulus categories (joy, anxiety, sadness, and neutral). ** p < 0.001.

As shown in Table 2 and Fig 1, joy ratings were significantly higher than other emotion ratings (fear/anxiety, disgust, and sadness; all p < 0.001, effect size r > 0.85) in joy stimulus condition. In anxiety condition, fear/anxiety ratings were significantly higher than other emotion ratings (all p < 0.001, effect size r > 0.85), and in sadness condition, sadness ratings were significantly higher than other emotion ratings (all p < 0.001, effect size r > 0.85). In neutral stimulus condition, joy ratings were significantly higher than other emotion ratings (all p < 0.001, effect size r > 0.5), but the overall magnitude of emotional responses was markedly lower than in other emotion conditions (in both self-reported ratings and facial expressions). These results indicate that each stimulus condition (except for the neutral stimuli) reliably elicited its intended target emotion as the predominant response.

**Table 2:** Wilcoxon signed-rank tests comparing predominant and other emotional responses in self-reported emotion ratings across stimulus conditions.

StimuliCondition	Comparison (Predominant vs. Other)	First Variable		Second Variable		z value	p value	Effect Size (r)
		Median	IQR	Median	IQR			
Joy	Joy vs. Anxiety/Fear	6.87	2.76	0.05	0.50	10.523	< 0.001	0.865
	Joy vs. Disgust	6.87	2.76	0.02	0.29	10.494	< 0.001	0.863
	Joy vs. Sadness	6.87	2.76	0.03	0.07	10.550	< 0.001	0.867
Neutral	Joy vs. Anxiety/Fear	0.54	1.58	0.09	0.20	7.142	< 0.001	0.587
	Joy vs. Disgust	0.54	1.58	0.02	0.04	8.287	< 0.001	0.681
	Joy vs. Sadness	0.54	1.58	0.03	0.06	8.071	< 0.001	0.663
Anxiety	Anxiety/Fear vs. Disgust	7.01	2.85	0.36	1.12	10.553	< 0.001	0.867
	Anxiety/Fear vs. Sadness	7.01	2.85	1.32	2.90	10.500	< 0.001	0.863
	Anxiety/Fear vs. Joy	7.01	2.85	0.01	0.05	10.553	< 0.001	0.867
Sadness	Sadness vs. Anxiety/Fear	7.32	2.31	2.60	3.68	10.506	< 0.001	0.864
	Sadness vs. Disgust	7.32	2.31	0.01	0.01	10.553	< 0.001	0.867
	Sadness vs. Joy	7.32	2.31	0.00	0.02	10.553	< 0.001	0.867

**Fig 2:**
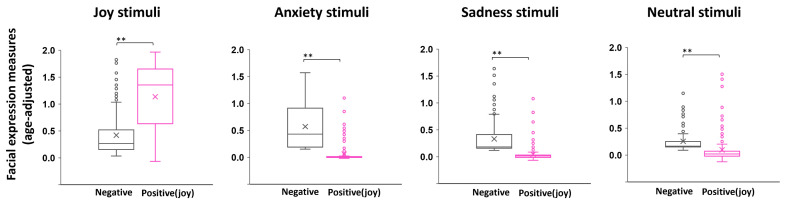
Boxplots showing medians, interquartile ranges, and individual data points comparing negative and positive facial expression measures across stimulus categories (joy, anxiety, sadness, and neutral). ** p < 0.001.

For facial expressions (Table 3 and Fig 2), positive facial expressions were significantly higher than negative in joy stimulus condition (p < 0.001, effect size, r = 0.735), whereas negative facial expressions were significantly higher than positive facial expressions in neutral (p < 0.001, r = 0.576), anxiety (p < 0.001, r = 0.825), and sadness conditions (p < 0.001, r = 0.829).

**Table 3:** Wilcoxon signed-rank tests comparing predominant and other emotional responses in facial expression measures across stimulus conditions.

StimuliCondition	Comparison (Predominant vs. Other)	First Variable		Second Variable		z Value	p Value	Effect Size (r)
		Median	IQR	Median	IQR			
Joy	Positive(Joy) vs. Negative	1.36	1.02	0.26	0.37	8.942	< .001	0.735
Neutral	Negative vs. Positive(Joy)	0.17	0.10	0.02	0.10	7.010	< .001	0.576
Anxiety	Negative vs. Positive(Joy)	0.43	0.72	0.01	0.02	10.042	< .001	0.825
Sadness	Negative vs. Positive(Joy)	0.18	0.25	0.01	0.05	10.081	< .001	0.829

### Sex differences

#### Facial expressions and self-reported emotion ratings.

Sex differences were examined for each stimulus emotion condition, separately for each response type using the Mann–Whitney U test. Prior to analyses, negative emotion ratings were derived as the highest score among the sadness, disgust, and fear/anxiety ratings for joy and neutral stimulus conditions. Given that these emotions did not emerge as predominant emotional responses in those conditions, this approach was unlikely to result in meaningful information loss while reducing analytical complexity. The Bonferroni correction was applied separately for self-reported ratings and facial expression measures, with two comparisons within each, resulting in an adjusted significance threshold of p < 0.025 (0.05/2).

**Fig 3:**
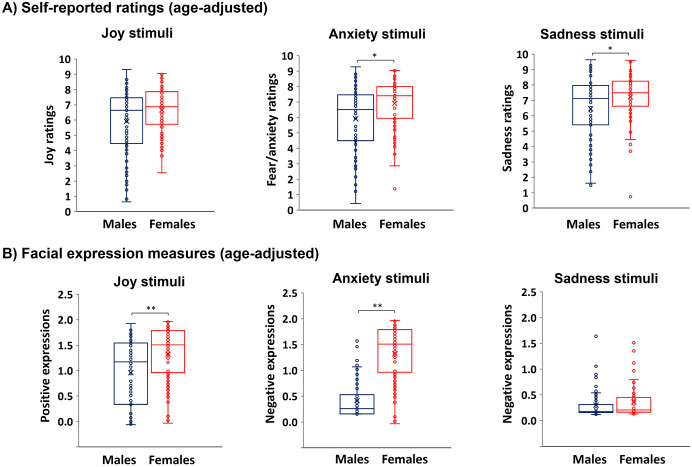
Boxplots showing medians, interquartile ranges, and individual data points comparing males and females in (A) self-reported emotion ratings and (B) facial expression measures for joy, anxiety and sadness stimulus conditions. * p < 0.025; ** p < 0.001.

As shown in Fig 3 and S5 Table in S1 File, in joy stimulus condition, females exhibited significantly higher positive facial expression scores than males (U = 1754.5, Z = –3.77, p < 0.001), while no sex differences were found in negative facial expression (U = 2309, Z = –1.643, p = 0.1) or self-rating scores (joy rating: U = 2256, Z = –1.847, p = 0.06; negative emotion rating: U = 2391.5, Z = –1.327, p = 0.184).

For anxiety stimuli, females showed significantly higher fear/anxiety ratings (U = 1890, Z = –3.25, p = 0.001) and negative facial expression scores than males (U = 1437, Z = –4.988, p < 0.001), whereas no sex differences were observed in joy ratings (U = 2400.5, Z = –1.293, p = 0.196) or positive facial expressions (U = 2701, Z = –0.14, p = 0.889).

For sadness condition, females reported significantly higher sadness ratings than males (U = 2092, Z = –2.476, p = 0.013), while no sex differences were found in joy ratings (U = 2728.5, Z = –0.035, p = 0.972) or in facial expression (positive facial expression: U = 2518.5, Z = –0.84, p = 0.401; negative facial expression: U = 2329.5, Z = –1.565, p = 0.118).

For neutral stimulus condition, females reported significantly higher joy ratings than males (U = 2132.5, Z = –2.321, p = 0.020), with no sex difference in negative emotion ratings (U = 2269.5, Z = –1.795, p = 0.073) or in facial expressions (positive facial expression: U = 2491.5, Z = –0.944, p = 0.345; negative facial expression: U = 2598.5, Z = –0.533, p = 0.594). Further analysis revealed that this effect was driven by two videos depicting familiar shopping districts (Neutral04 and Neutral08), for which females showed significantly higher joy ratings than males (Neutral04: U = 2220.5, Z = –1.983, p = 0.047; Neutral08: U = 2129.5, Z = –2.332, p = 0.020). No significant sex differences were observed for the remaining neutral clips (all p > 0.12; see S6 Table in S1 File). After removing the two clips, the sex difference in joy ratings was no longer significant (U = 2289.5, Z = –1.718, p = 0.086), suggesting that the observed effect may be driven by stimulus-specific familiarity and positive associations with well-known locations rather than a general sex difference in emotional responses.

In supplementary analyses, results for individual joy, sadness, fear and disgust facial expression measures are provided in Supplementary Table S7 in S1 File. These findings showed a similar pattern to those obtained using the valence-based facial expression scores.

#### Self-reported affective ratings.

**Fig 4:**
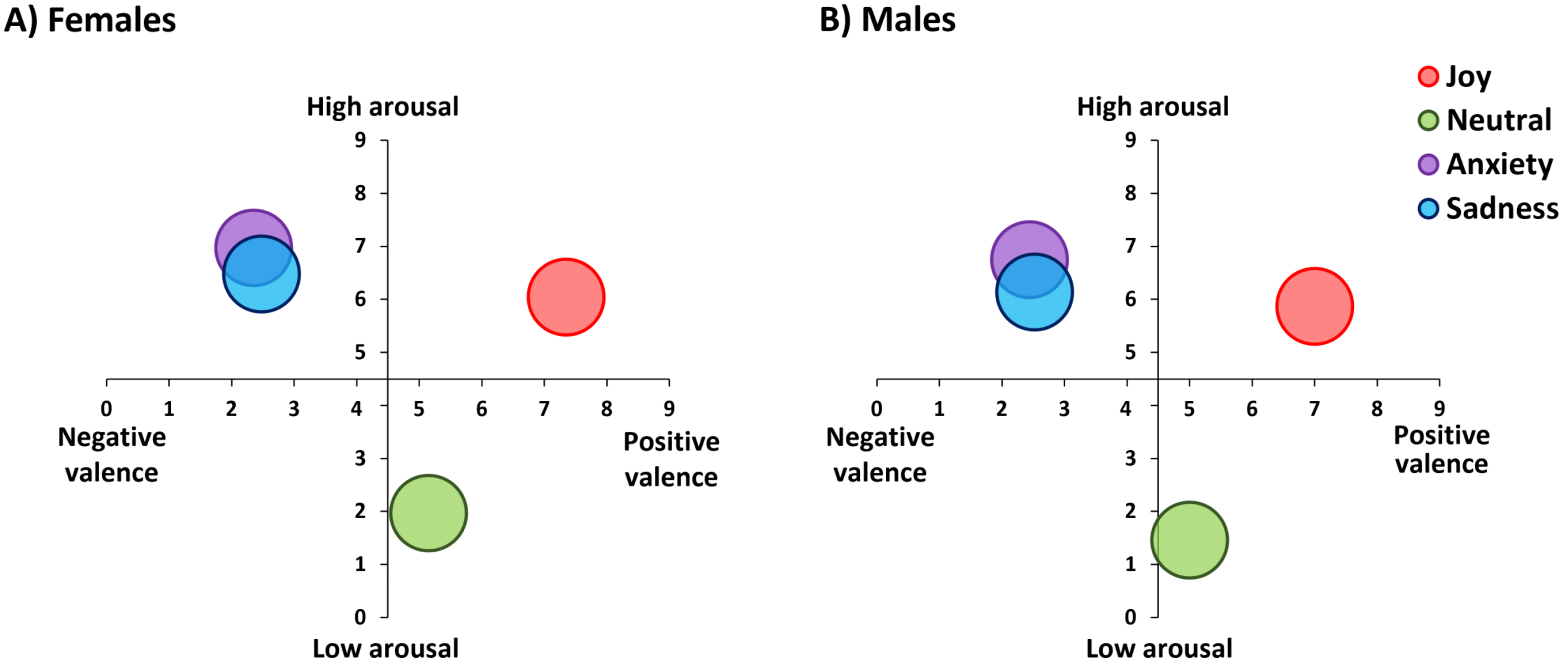
Mean valence (x-axis) and arousal (y-axis) self-ratings for each stimulus category (joy, anxiety, sadness, neutral) are shown separately for females (A) and males (B).

The affective dimension ratings (valence and arousal) showed distinct valence–arousal profiles for each stimulus emotion category (Fig 4). To examine sex differences, Mann–Whitney U tests were conducted for arousal and valence ratings across stimulus emotion categories (S8 Table in S1 File), with Bonferroni correction applied (p < 0.025).

As shown in Fig 4, joy stimuli showed high positive valence (M: Median = 7, IQR = 1.37; F: Median = 7.35, IQR = 1.31) and high arousal (M: Median = 5.87, IQR = 2.62; F: Median = 6.04, IQR = 1.66) in both males and females, with no significant sex differences in either valence (U = 2314.5, Z = −1.622, p = 0.105) or arousal (U = 2250, Z = −1.87, p = 0.062).

Anxiety stimuli elicited negative valence (M: Median = 2.44, IQR = 1.18; F: Median = 2.35, IQR = 1.46) and high arousal (M: Median = 6.75, IQR = 2.21; F: Median = 6.97, IQR = 1.73) in both sexes. Females reported significantly higher arousal than males (U = 1955, Z = −3.001, p = 0.003), whereas no sex difference was found in valence ratings (U = 2259.5, Z = −1.833, p = 0.067).

Sadness stimuli also elicited negative valence (M: Median = 2.52, IQR = 1.38; F: Median = 2.48, IQR = 1.63) and high arousal (M: Median = 6.14, IQR = 2.84; F: Median = 6.48, IQR = 2.32), although arousal levels were lower than those elicited by anxiety stimuli. Similar to anxiety stimuli, females reported significantly higher arousal than males (U = 2142.5, Z = −2.282, p = 0.022), while valence ratings did not differ by sex (U = 2348, Z = −1.494, p = 0.135).

Neutral stimuli exhibited mid-range valence (M: Median = 5, IQR = 0.13; F: Median = 5.15, IQR = 0.14) and low arousal (M: Median = 1.46, IQR = 1.32; F: Median = 1.97, IQR = 1.54), with no significant sex differences in either dimension (arousal: U = 2565, Z = −0.662, p = 0.508; valence: U = 2564.5, Z = −0.664, p = 0.507).

### Correlations between self-reported emotion ratings and facial expressions

**Fig 5:**
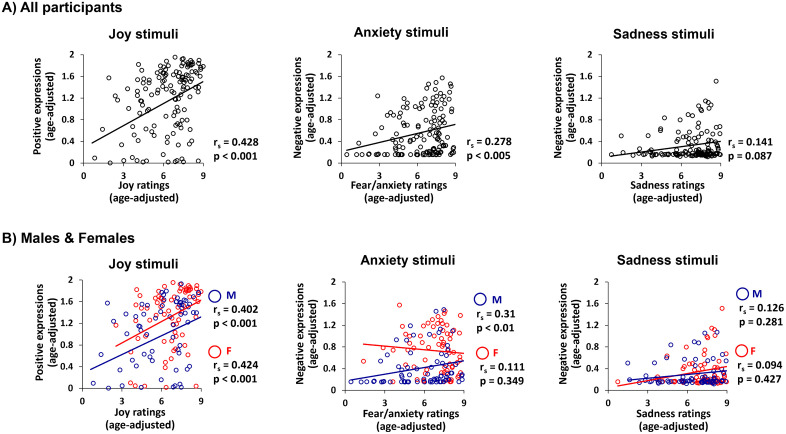
Scatter plots with regression lines comparing predominant emotional self-reported emotion ratings (x-axis) against predominant facial expression measures (y-axis) in joy, anxiety and sadness stimulus conditions for all participants (A) and for males and females (B). Spearman’s rank correlation coefficients and corresponding p-values are shown in the bottom right of each plot. Bonferroni correction was applied to adjust for multiple comparisons (p < 0.05/3 = 0.016).

Spearman’s rank correlations (ρ) were computed between self-reported emotion ratings and facial expression measures for the entire sample (N = 148) and separately for male and female participants, with Bonferroni correction applied to adjust for multiple comparisons (p < 0.05/3 = 0.016). Correlation analyses focused on predominant emotional responses (e.g., joy ratings, fear/anxiety ratings, and sadness ratings) elicited by joy, anxiety, and sadness stimuli, respectively; the neutral condition was excluded because no significant predominant emotional responses were observed (Fig 5).

For joy stimuli, significant positive correlations were observed in the entire sample (ρ = 0.428, p < 0.001) and in both males (ρ = 0.402, p < 0.001) and females (ρ = 0.424, p < 0.001). For anxiety stimuli, significant positive correlations were observed in the entire sample (ρ = 0.278, p = 0.001) and males (ρ = 0.310, p = 0.007), but not in females (ρ = 0.111, p = 0.349). For sadness stimuli, no significant correlations were found in the entire sample (ρ = 0.141, p = 0.087), males (ρ = 0.126, p = 0.281), or females (ρ = 0.094, p = 0.427).

## Discussion

In this study, we examined sex differences in self-reported emotional ratings and facial expression responses to dynamic emotional video stimuli (joy, anxiety, sadness, and neutral stimuli) in Korean adults, a population in which emotional responses to such stimuli and their potential sex differences in these responses, have been relatively understudied compared with Western populations. By addressing this gap, the present study provides culturally specific evidence and a basis for subsequent research. Furthermore, prior research on sex differences in emotional responding has largely examined subjective experience and facial expression in isolation [18,21], or has been constrained by relatively small sample sizes [12]. The present study contributes to the literature by integrating self-reported experience and continuous facial expression measures in a comparatively large sample. The main findings of this study are described below.

First, the emotional video stimuli used in this study elicited distinct arousal and valence profiles. Self-reported affective dimension ratings revealed that joy stimuli were characterized by high arousal and positive valence, whereas anxiety and sadness stimuli were characterized by high arousal and negative valence, clearly distinguishing them from neutral stimuli, which showed mid-range valence and low arousal. These configurations closely align with established affective space models and provide empirical support for the validity of our stimuli [32].

Second, we found convergence between facial expression indices and subjective experiences (i.e., self-reported ratings) with respect to predominant responses. By contrasting positive and negative facial expression measures, we identified the dominant facial responses across different emotion categories, with joy stimuli eliciting significantly stronger positive expressions, whereas anxiety and sadness stimuli eliciting significantly stronger negative expressions. These facial expression patterns were consistent with self-reported ratings, which showed the highest joy ratings for joy stimuli, the highest sadness ratings for sadness stimuli, and the highest fear/anxiety ratings for anxiety stimuli. This multimodal convergence in the predominant responses provides supportive evidence for the validity of our emotion induction paradigm and analytic approach and suggests that continuous facial expression measures may serve as an objective complement to self-reported measures.

Third, our results indicate that sex differences in emotional responding vary both across emotion categories and between response measures, rather than showing a uniform pattern across emotions and response types. In the joy stimuli condition, females exhibited stronger joy-related facial expressions than males whereas no sex difference emerged in self-reported joy ratings. This pattern is consistent with previous findings suggesting that females tend to display greater facial expressivity for positive emotions, possibly reflecting socialization processes that encourage expressive communication of affiliative states [18,33,34]. In the anxiety stimuli condition, sex differences were observed in both response measures, with females exhibiting stronger negative facial expressions as well as higher fear/anxiety ratings than males. These findings align with neuroimaging evidence demonstrating that females often exhibit greater physiological and neurobiological reactivity than males, such as greater amygdala and limbic activation, to high-arousal negative stimuli compared to males [35,36]. For sadness stimuli, sex differences were observed only in self-reported ratings, with females reporting higher sadness ratings than males while no corresponding difference emerged in facial expressions. This dissociation may reflect cultural and regulatory norms surrounding sadness expression in adults, where sadness is often internalized rather than overtly expressed [37]. Notably, our facial expression indices in the sadness condition were relatively low for both males and females compared with those for joy and anxiety conditions, which may have affected the likelihood of detecting expressive differences.

We further observed that the associations between facial expression indices and self-reported ratings varied across emotion categories and sexes. In the joy condition, strong positive correlations were found between joy self-ratings and positive facial expression scores in both sexes. The findings are consistent with prior research suggesting that positive emotions, particularly joy, are more likely to be overtly and consistently expressed facially, reflecting an evolutionarily conserved communicative function [38,39], which may enhance the detectability of joy-related facial responses. Moreover, happy facial expressions have been shown to be identified with relatively high consistency compared to other emotions in Korean adults, which may contribute to the strong concordance between subjective and facial measures in this study [40]. In contrast, in the sadness condition, no significant correlations between sadness self-ratings and negative facial expression scores were found in any group. In the present study, sadness-related facial expressions were generally low in intensity and less dynamically expressed, particularly under passive viewing conditions. This may have resulted in restricted variance and reduced detectability in automated facial metrics [41,42], thereby limiting covariance with self-reported sadness. The findings suggest that stronger or more personally salient sadness-inducing stimuli may be required to elicit measurable facial responses in future studies. In the anxiety condition, only males showed significant correlation between fear ratings and negative facial expressions. This pattern may reflect sex differences in regulatory strategies for facial expressivity under threat-related conditions, where males show closer alignment between subjective fear and facial expression while females may attenuate expressive cues despite elevated subjective arousal [43,44].

Finally, certain neutral video stimuli (Neutral04 and Neutral08) elicited significantly higher joy ratings in females than in males. This finding suggests that ostensibly neutral stimuli may unintentionally evoke affective responses depending on participant characteristics and contextual interpretation, potentially compromising the reliability and interpretability of emotion induction paradigms. Future studies should therefore ensure the validity of neutral stimuli through careful stimulus selection and validation procedures.

In conclusion, this study provides culturally specific data on facial expressions, emotional self-ratings, and sex-related differences in the emotional responding in a Korean adult sample, helping to address the relative lack of emotion validation studies in non-Western populations. Furthermore, to our knowledge, continuous and automated analyses of spontaneous and naturalistic facial expressions in Korean adults remain limited. Most prior studies of Korean facial expressions have relied on static and posed expressions [40,45,46], which may constrain ecological validity given that real-life facial expressions are inherently dynamic. The present study extends the literature by examining dynamic facial signals across the entire duration of stimulus presentation. Our findings provide evidence for the feasibility of continuous facial expression analysis in a Korean sample and suggest that this approach can capture temporally unfolding emotional responses in a systematic manner.

Several limitations warrant consideration. Sadness stimuli elicited relatively weak facial responses, indicating that more potent or personally salient sadness-inducing materials may be required in future work. Moreover, as facial expressions alone may not fully capture emotional responding, integrating physiological and neural measures would allow for a more comprehensive multimodal assessment. Future studies extending this paradigm to clinical populations may further clarify how discrepancies between subjective experience and expressive behavior relate to diagnosis or prognosis, thereby enhancing the translational value of video-based emotional paradigms.

## Supporting information

S1 File Supplementary materials. This file contains the supplementary figure and tables supporting the study, including the experimental protocol for emotional video stimuli, Facial Action Units used for facial expression analysis, reliability estimates between iMotions and a human rater, descriptive statistics of age-controlled self-reported emotion ratings and facial expression measures across stimulus conditions, normality test results, and sex-difference analyses for emotion ratings, facial expressions, arousal, and valence.
(DOCX)
