# Supplementary material for: Divergence between facial expressions and self-reported emotions: Sex differences in responses to video-based emotional stimuli
Source: PLoS One. 2026 Jun 29;21(6):e0352759. doi: 10.1371/journal.pone.0352759 (PMC13313359; doi:10.1371/journal.pone.0352759)
Supplement: S1 File — This file contains the supplementary figure and tables supporting the study, including the experimental protocol for emotional video stimuli, Facial Action Units used for facial expression analysis, reliability estimates between iMotions and a human rater, descriptive statistics of age-controlled self-reported emotion ratings and facial expression measures across stimulus conditions, normality test results, and sex-difference analyses for emotion ratings, facial expressions, arousal, and valence. (DOCX) [file pone.0352759.s001.docx]

**S1 File. Supplementary Materials**

**
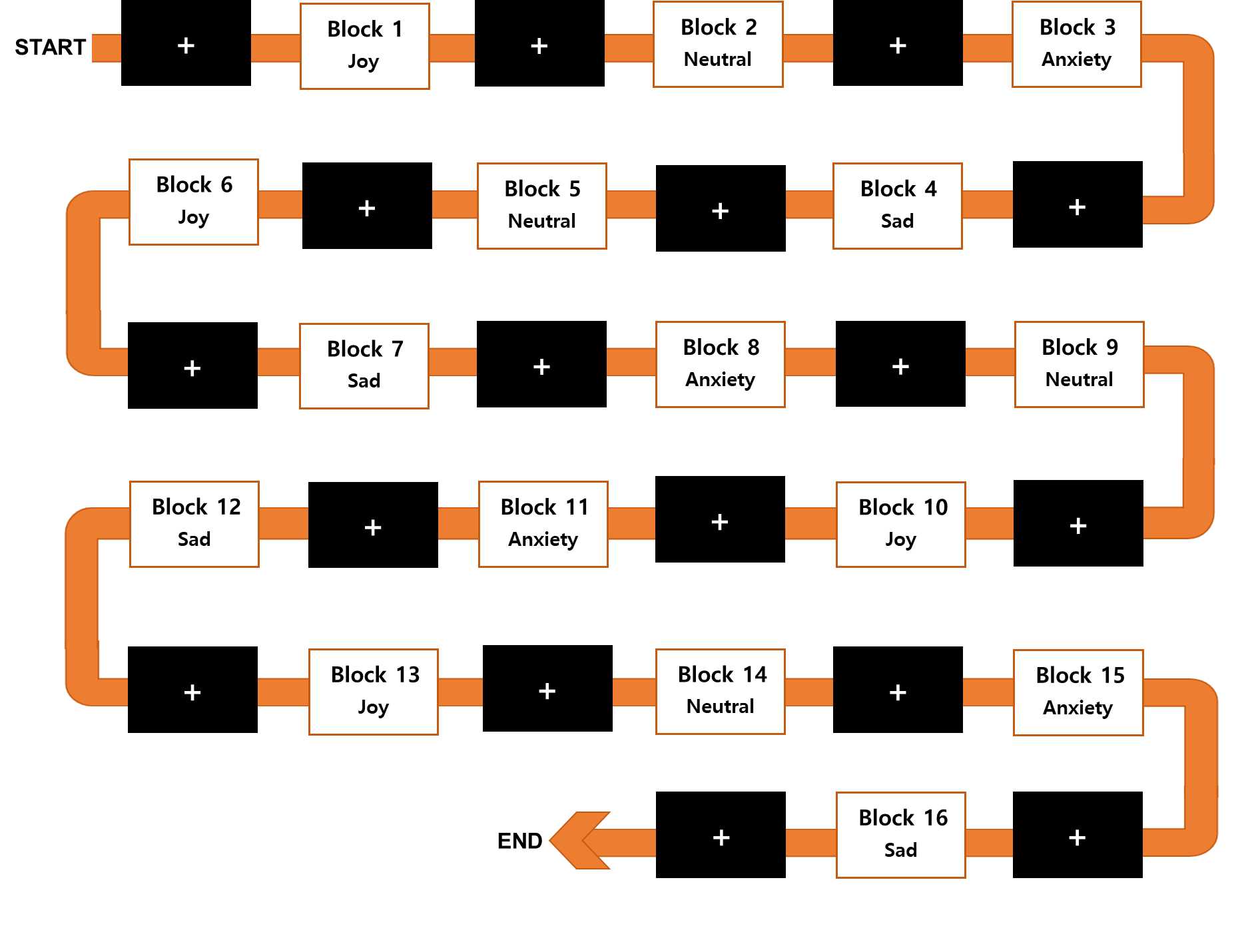
**

**S1 Fig.** Participants were exposed to emotional video stimuli designed to elicit facial muscle movements. The audio-visual materials were delivered on a laptop monitor with auditory output through headphones to enhance naturalistic engagement. The experimental protocol began with a 30-second fixation cross and then proceeded to 16 stimulus blocks, each consisting of 30 seconds of emotional content followed by a 10-second fixation period (total duration: 670 seconds, 30 fps). A total of 16 distinct stimulus blocks were generated from the four emotional categories (Joy, sadness, anxiety-inducing, and neutral), with each block containing a different combination of video clips. These 16 blocks were divided into two sequences of eight blocks, and both sequences were presented in a pseudo-randomized order. Each 30-second block contained two to three short clips drawn from comedy programs, televised broadcasts, and online video platforms (YouTube). For ethical compliance and copyright considerations, no original screenshots from the video materials are displayed in this manuscript; instead, the stimulus blocks are represented schematically using labeled boxes/placeholders. Participants viewed the original unaltered footage during the experiment. Joy stimuli were compiled from Korean entertainment programs such as “Running Man”, “Infinite Challenge”, “Where Are You Going, Dad?”, and “Real Men”, along with humorous YouTube compilations including “Best Babies Laughing Video Compilation” and content from “TuATuJi TV.” Sadness stimuli were sourced from “Documentary Hospice 24 Hours” and “Human Documentary: Love”, capturing emotionally intense scenes of family members crying while saying farewell to terminally ill parents, as well as parents expressing grief beside critically ill children. Anxiety-inducing stimuli consisted of YouTube footage of animal attacks, natural disasters (earthquakes and tsunamis), gas explosions, wartime airstrikes, and dashboard camera recordings captured from inside vehicles evacuating along roads engulfed by rapidly advancing wildfires, as well as accident clips from extreme sports. Neutral stimuli featured street-view footage of everyday urban environments, presented with natural ambient sounds and no added background music to minimize emotional induction.

**S1 Table.** Facial Action Units (AUs)

| **Facial movement** | **Corresponding  Action Unit (AU)** |
| --- | --- |
| Brow Raise | AU2 |
| Brow Furrow | AU4 |
| Eye Widen | AU5 |
| Cheek Raise | AU6 |
| Lid Tighten | AU7 |
| Nose Wrinkle | AU9 |
| Upper Lip Raise | AU10 |
| Dimpler | AU14 |
| Lip Corner Depressor | AU15 |
| Chin Raise | AU17 |
| Lip Pucker | AU18 |
| Lip Stretch | AU20 |
| Lip Press | AU24 |
| Mouth Open | AU25 |
| Jaw Drop | AU26 |
| Lip Suck | AU28 |
| Eye Closure | AU43 |

**S2 Table.** Reliability of facial expression measures between iMotions and a human rater

| **Facial Expression** | **ICC (1,2)** | **95% CI** | **p Value** |
| --- | --- | --- | --- |
| Joy | 0.938 | 0.907-0.959 | < .001 |
| Sadness | 0.716 | 0.573-0.811 | < .001 |
| Fear | 0.718 | 0.576-0.812 | < .001 |
| Disgust | 0.727 | 0.589-0.818 | < .001 |
| Surprise | 0.740 | 0.610-0.827 | < .001 |
| Negative | 0.846 | 0.769-0.898 | < .001 |

**Supplementary Table S3.** Descriptive statistics (median, IQR) of age-controlled self-reported emotion ratings and iMotions facial expressions across stimulus conditions (N=148)

| **Stimulus Condition** | **Age-Controlled Variable** | | **Median** | **IQR** |
| --- | --- | --- | --- | --- |
| Joy | Emotion Rating | Joy | 6.87 | 2.76 |
|  |  | Sadness | 0.03 | 0.07 |
|  |  | Fear/anxiety | 0.05 | 0.50 |
|  |  | Disgust | 0.02 | 0.29 |
|  |  | Negative | 0.26 | 0.37 |
|  | Facial Expression | Positive (Joy) | 1.36 | 1.02 |
|  |  | Negative | 0.26 | 0.37 |
| Neutral | Emotion Rating | Joy | 0.54 | 1.58 |
|  |  | Sadness | 0.03 | 0.06 |
|  |  | Fear/anxiety | 0.09 | 0.20 |
|  |  | Disgust | 0.02 | 0.04 |
|  |  | Negative | 0.02 | 0.18 |
|  | Facial Expression | Positive (Joy) | 0.02 | 0.10 |
|  |  | Negative | 0.17 | 0.10 |
| Sadness | Emotion Rating | Joy | 0.00 | 0.02 |
|  |  | Sadness | 7.32 | 2.31 |
|  |  | Fear/anxiety | 2.60 | 3.68 |
|  |  | Disgust | 0.01 | 0.01 |
|  | Facial Expression | Positive (Joy) | 0.01 | 0.05 |
|  |  | Negative | 0.18 | 0.25 |
| Anxiety | Emotion Rating | Joy | 0.01 | 0.05 |
|  |  | Sadness | 1.32 | 2.90 |
|  |  | Fear/anxiety | 7.01 | 2.85 |
|  |  | Disgust | 0.36 | 1.12 |
|  | Facial Expression | Positive (Joy) | 0.01 | 0.02 |
|  |  | Negative | 0.43 | 0.72 |

**S4 Table.** Kolmogorov-Smirnov tests of age-controlled variables across stimulus conditions (N=148)

| **Stimulus Condition** | **Age Controlled Variable** | | **K-S Statistic (D)** | **p Value** | **Normality** |
| --- | --- | --- | --- | --- | --- |
| Joy | Emotion Rating | Joy | 0.129 | < .001 | Violated |
|  |  | Sadness | 0.409 | < .001 | Violated |
|  |  | Fear/anxiety | 0.304 | < .001 | Violated |
|  |  | Disgust | 0.321 | < .001 | Violated |
|  |  | Arousal | 0.093 | < .005 | Violated |
|  |  | Valence | 0.051 | 0.200 | Met |
|  | Facial Expression | Positive (Joy) | 0.146 | < .001 | Violated |
|  |  | Negative | 0.208 | < .001 | Violated |
| Neutral | Emotion Rating | Joy | 0.191 | < .001 | Violated |
|  |  | Sadness | 0.447 | < .001 | Violated |
|  |  | Fear/anxiety | 0.349 | < .001 | Violated |
|  |  | Disgust | 0.478 | < .001 | Violated |
|  |  | Arousal | 0.200 | < .001 | Violated |
|  |  | Valence | 0.277 | < .001 | Violated |
|  | Facial Expression | Positive (Joy) | 0.347 | < .001 | Violated |
|  |  | Negative | 0.301 | < .001 | Violated |
| Sadness | Emotion Rating | Joy | 0.501 | < .001 | Violated |
|  |  | Sadness | 0.144 | < .001 | Violated |
|  |  | Fear/anxiety | 0.103 | < .001 | Violated |
|  |  | Disgust | 0.413 | < .001 | Violated |
|  |  | Arousal | 0.115 | < .001 | Violated |
|  |  | Valence | 0.101 | < .001 | Violated |
|  | Facial Expression | Positive (Joy) | 0.383 | < .001 | Violated |
|  |  | Negative | 0.274 | < .001 | Violated |
| Anxiety | Emotion Rating | Joy | 0.395 | < .001 | Violated |
|  |  | Sadness | 0.159 | < .001 | Violated |
|  |  | Fear/anxiety | 0.140 | < .001 | Violated |
|  |  | Disgust | 0.234 | < .001 | Violated |
|  |  | Arousal | 0.135 | < .001 | Violated |
|  |  | Valence | 0.087 | < .01 | Violated |
|  | Facial Expression | Positive (Joy) | 0.425 | < .001 | Violated |
|  |  | Negative | 0.166 | < .001 | Violated |

| **Stimulus Condition** | **Response type** | | **Males (N=75)** | | **Females(N=73)** | | **Statistics** | | |
| --- | --- | --- | --- | --- | --- | --- | --- | --- | --- |
|  |  |  | **Median** | **IQR** | **Median** | **IQR** | **Mann-Whitney U** | **z Value** | **p Value** |
| Joy | Emotion Rating | Joy | 6.64 | 3 | 6.87 | 2.14 | 2256 | -1.847 | 0.065 |
|  |  | Negative | 0.21 | 0.75 | 0.77 | 1.08 | 2391.5 | -1.327 | 0.184 |
|  | Facial Expression | Positive (Joy) | 1.17 | 1.2 | 1.32 | 0.83 | 1754.5 | -3.77 | < .001 |
|  |  | Negative | 0.23 | 0.26 | 0.47 | 0.54 | 2309 | -1.643 | 0.100 |
| Neutral | Emotion Rating | Joy | 0.38 | 1.39 | 0.94 | 1.84 | 2132.5 | -2.321 | 0.02 |
|  |  | Negative | 0.02 | 0.06 | 0.39 | 0.56 | 2269.5 | -1.795 | 0.073 |
|  | Facial Expression | Positive (Joy) | 0.01 | 0.09 | 0.13 | 0.11 | 2491.5 | -0.944 | 0.345 |
|  |  | Negative | 0.17 | 0.08 | 0.25 | 0.11 | 2598.5 | -0.533 | 0.594 |
| Anxiety | Emotion Rating | Joy | 0.01 | 0.05 | 0 | 0.05 | 2400.5 | -1.293 | 0.196 |
|  |  | Fear/Anxiety | 6.51 | 2.96 | 7.4 | 2.05 | 1890 | -3.25 | 0.001 |
|  | Facial Expression | Positive (Joy) | 0.01 | 0.01 | 0.07 | 0.02 | 2701 | -0.14 | 0.889 |
|  |  | Negative | 0.26 | 0.37 | 0.73 | 0.7 | 1437 | -4.988 | < .001 |
| Sadness | Emotion Rating | Joy | 0.00 | 0.02 | 0.00 | 0.02 | 2728.5 | -0.035 | 0.972 |
|  |  | Sadness | 7.11 | 2.56 | 7.49 | 1.63 | 2092 | -2.476 | 0.013 |
|  | Facial Expression | Positive (Joy) | 0.01 | 0.05 | 0.05 | 0.06 | 2518.5 | -0.84 | 0.401 |
|  |  | Negative | 0.17 | 0.16 | 0.36 | 0.29 | 2329.5 | -1.565 | 0.118 |

**S5 Table**. Mann-Whitney U tests for sex differences in self-reported emotion ratings and facial expressions across stimulus conditions

*Notes.* Bonferroni correction was applied separately for self-reported ratings and facial expression measures (p < 0.025).

**S6 Table.** Mann-Whitney U tests for sex differences in joy ratings across individual neutral stimuli video clips

| **Emotional  Self-rating** | **Male (N=75)** | | **Female (N=73)** | | **Statistic** | | |
| --- | --- | --- | --- | --- | --- | --- | --- |
|  | **Median** | **IQR** | **Median** | **IQR** | **Mann- Whitney U** | **Z** | **p** |
| Neutral01-Joyful | 0.36 | 2.30 | 0.77 | 2.85 | 2340.0 | -1.525 | 0.127 |
| Neutral02-Joyful | 0.20 | 0.98 | 0.43 | 2.17 | 2372.0 | -1.402 | 0.161 |
| Neutral03-Joyful | 0.35 | 1.60 | 0.38 | 2.43 | 2631.0 | -0.409 | 0.683 |
| Neutral04-Joyful | 0.25 | 2.19 | 0.57 | 3.20 | 2220.5 | -1.983 | 0.047 |
| Neutral05-Joyful | 0.26 | 1.16 | 0.32 | 2.09 | 2647.0 | -0.347 | 0.728 |
| Neutral06-Joyful | 0.06 | 0.48 | 0.13 | 1.58 | 2423.5 | -1.205 | 0.228 |
| Neutral07-Joyful | 0.24 | 1.57 | 0.33 | 2.38 | 2557.0 | -0.692 | 0.489 |
| Neutral08-Joyful | 0.20 | 2.41 | 1.21 | 3.50 | 2129.5 | -2.332 | 0.020 |

**S7 Table**. Mann-Whitney U tests for sex differences in discrete facial expression metrics (joy, sadness, fear, and disgust) across stimulus conditions

| **Stimulus Condition** | **Facial Expression** | | **Males (N=75)** | | | | **Females(N=73)** | | | | **Statistics** | | | | | |
| --- | --- | --- | --- | --- | --- | --- | --- | --- | --- | --- | --- | --- | --- | --- | --- | --- |
|  |  |  | **Median** | | **IQR** | | **Median** | | **IQR** | | **Mann-Whitney U** | | **z Value** | | **p Value** | |
| Joy | Joy | 1.17 | | 1.2 | | 1.32 | | 0.83 | | 1754.5 | | -3.77 | | < .001 | |  |
|  | Sadness | 0.01 | | 0.01 | | 0.01 | | 0.05 | | 2463 | | -1.05 | | 0.292 | |  |
|  | Fear | 0.00 | | 0.01 | | 0.01 | | 0.02 | | 2348 | | -1.49 | | 0.135 | |  |
|  | Disgust | 0.16 | | 0.14 | | 0.16 | | 0.18 | | 2620 | | -0.451 | | 0.652 | |  |
| Neutral | Joy | 0.01 | | 0.09 | | 0.13 | | 0.11 | | 2491.5 | | -0.944 | | 0.345 | |  |
|  | Sadness | 0.01 | | 0.02 | | 0.02 | | 0.02 | | 2472 | | -1.018 | | 0.309 | |  |
|  | Fear | 0.00 | | 0.03 | | 0.01 | | 0.03 | | 2357 | | -1.459 | | 0.144 | |  |
|  | Disgust | 0.16 | | 0.02 | | 0.16 | | 0.02 | | 2266 | | -1.808 | | 0.071 | |  |
| Anxiety | Joy | 0.01 | | 0.01 | | 0.07 | | 0.02 | | 2701 | | -0.14 | | 0.889 | |  |
|  | Sadness | 0.02 | | 0.07 | | 0.14 | | 0.36 | | 1247 | | -5.717 | | < .001 | |  |
|  | Fear | 0.00 | | 0.04 | | 0.03 | | 0.08 | | 1882 | | -3.281 | | 0.001 | |  |
|  | Disgust | 0.16 | | 0.09 | | 0.19 | | 0.16 | | 2655 | | -0.316 | | 0.752 | |  |
| Sadness | Joy | 0.01 | | 0.05 | | 0.05 | | 0.06 | | 2518.5 | | -0.84 | | 0.401 | |  |
|  | Sadness | 0.01 | | 0.02 | | 0.02 | | 0.06 | | 2190 | | -2.100 | | 0.036 | |  |
|  | Fear | 0.00 | | 0.01 | | 0.01 | | 0.02 | | 2363 | | -1.436 | | 0.151 | |  |
|  | Disgust | 0.16 | | 0.03 | | 0.16 | | 0.03 | | 2618 | | -0.458 | | 0.647 | |  |

*Notes.* Bonferroni correction was applied (p < 0.0125).

**S8 Table.** Mann-Whitney U tests for sex differences in arousal and valence ratings across stimulus conditions

| **Stimulus Condition** | **Response type** | **Male (N=75)** | | **Female (N=73)** | | **Statistics** | | |  |
| --- | --- | --- | --- | --- | --- | --- | --- | --- | --- |
|  |  | **Median** | **IQR** | **Median** | **IQR** | **Mann-Whitney U** | **z value** | **p value** | |
| Joy | Arousal | 5.87 | 2.62 | 6.04 | 1.66 | 2250 | -1.870 | 0.062 | |
|  | Valence | 7.00 | 1.37 | 7.35 | 1.31 | 2314.5 | -1.622 | 0.105 | |
| Neutral | Arousal | 1.46 | 1.32 | 1.97 | 1.54 | 2565 | -0.662 | 0.508 | |
|  | Valence | 5.00 | 0.13 | 5.15 | 0.14 | 2564.5 | -0.664 | 0.507 | |
| Anxiety | Arousal | 6.75 | 2.21 | 6.97 | 1.73 | 1955 | -3.001 | 0.003 | |
|  | Valence | 2.44 | 1.18 | 2.35 | 1.46 | 2259.5 | -1.833 | 0.067 | |
| Sadness | Arousal | 6.14 | 2.84 | 6.48 | 2.32 | 2142.5 | -2.282 | 0.022 | |
|  | Valence | 2.52 | 1.38 | 2.48 | 1.63 | 2348 | -1.494 | 0.135 | |

*Notes.* Bonferroni correction was applied separately for arousal and valence ratings (p < 0.025).
